# Fingerprinting of Proteases, Protease Inhibitors and Indigenous Peptides in Human Milk

**DOI:** 10.3390/nu15194169

**Published:** 2023-09-27

**Authors:** Martin Nørmark Thesbjerg, Søren Drud-Heydary Nielsen, Ulrik Kræmer Sundekilde, Nina Aagaard Poulsen, Lotte Bach Larsen

**Affiliations:** 1 Department of Food Science, Aarhus University, Agro Food Park 48, DK-8200 Aarhus, Denmark; mnt@food.au.dk (M.N.T.); nina.poulsen@food.au.dk (N.A.P.); 2 Sino-Danish College (SDC), University of Chinese Academy of Sciences, Huairou District, Beijing 101408, China

**Keywords:** human milk, proteomics, proteases, peptidases, bioactive peptides

## Abstract

The presence of proteases and their resulting level of activity on human milk (HM) proteins may aid in the generation of indigenous peptides as part of a pre-digestion process, of which some have potential bioactivity for the infant. The present study investigated the relative abundance of indigenous peptides and their cleavage products in relation to the abundance of observed proteases and protease inhibitors. The proteomes and peptidomes in twelve HM samples, representing six donors at lactation months 1 and 3, were profiled. In the proteome, 39 proteases and 29 protease inhibitors were identified in 2/3 of the samples. Cathepsin D was found to be present in higher abundance in the proteome compared with plasmin, while peptides originating from plasmin cleavage were more abundant than peptides from cathepsin D cleavage. As both proteases are present as a system of pro- and active- forms, their activation indexes were calculated. Plasmin was more active in lactation month 3 than month 1, which correlated with the total relative abundance of the cleavage product ascribed to plasmin. By searching the identified indigenous peptides in the milk bioactive peptide database, 283 peptides were ascribed to 10 groups of bioactivities. Antimicrobial peptides were significantly more abundant in month 1 than month 3; this group comprised 103 peptides, originating from the β-CN C-terminal region.

## 1. Introduction

Human milk (HM) is an evolutionary form of optimized nutrition for infants during early life. Therefore, HM has been vividly studied to identify the key components vital for supporting the development and growth of the infant [1,2,3,4]. Compared with bovine milk (BM), the protein content in HM is lower (approximately 1% *w*/*v* in HM vs. 3.4% *w*/*v* in BM) [5], and the whey:casein ratio is different, for BM approximately 1:4 and for HM approximately 3:2 [6]. Even though the total protein content in HM has been shown to vary a lot between individual mothers [7,8,9], an overall trend of a decrease in the protein content over the lactation period was shown, in addition to an increase in the whey:casein ratio over the lactation period (45:55 to 97:3) [7,8]. This is also reflected in compositional changes in individual milk proteins with the lactation stage [9,10]. 

HM is, to some extent, pre-digested by indigenous proteases within the mammary gland [11,12], which continues after ingestion by the infant [13], which can even degrade human κ-CN [14]. This pre-digestion of the HM proteins was suggested to aid the infant in the digestion of proteins and, thus, increase the nutritional uptake by the infant. A beneficial effect of this pre-digestion is also the generation of bioactive peptides, where a wide variety of these are known [15]. Furthermore, the profile of bioactive peptides is also known to differ between term and pre-term infants [16].

Mass spectrometry (MS)-based methods have allowed direct detection and determination of the generated indigenous peptides occurring in HM [11,12,13]. On the other side, detection of the array of proteases themselves, as well as their activators and inhibitors, has mainly been carried out by enzymatic and/or immunological assays [17,18], and only rarely by direct MS detection [19]. In some of the peptidome studies, the proteolytic enzyme was predicted based on the identified cleavage sites using tools such as Proteasix [20], PROSPER [21], enzyme predictor [22] or MEROPS [23]. MS has thus been shown to be a powerful method for the identification and quantification of naturally occurring peptides in HM, where the peptides were used to predict the responsible enzymes. Many of the enzymes were compared with BM and found to be of known importance in BM [19]. Identified proteases in HM comprise plasmin [12,17,24,25,26,27], cathepsins B, D [16,17,18], elastase [12,16,18,28], trypsin [28,29], kallikreins 6 and 11 [12], a range of amino- and carboxypeptidases [12,17], as well as matrix metalloproteinases (MMP) [30,31]. Most of these are regulated by a series of activators and inhibitors, which are also found in HM [17,32], e.g., urokinase [27,33], α-2-antiplasmin (SERPINF2) is known to inhibit plasmin, amongst others [17]. Several milk proteases are present mainly as proenzymes and, therefore, require activation and may not be active at the same level in all donors [28,29]. Major zymogen/enzyme systems known from HM are plasmin [24,26], cathepsin D, kallikrein and presumably other protease systems, including some of the other cathepsins, but their major milk form has not been studied in detail. In the case of plasminogen, in milk it is known to comprise both inactive plasminogen and active plasmin, the propeptide and the activation peptide thus need to be removed for activation [34], while procathepsin D is activated through the removal of an N-terminal activation peptide by a combination of autoactivation at an acidic pH and other proteases [35,36].

This study aimed to investigate the interplay between the present proteases, their inhibitors and naturally occurring peptides in HM using state-of-the-art nanoLC-MS/MS timsTOF Pro II. With this study, the goal was to investigate how the abundance of proteases, their activation degrees and inhibitors, related to the abundance of indigenous peptides, varied between donor milk samples in early lactation (months 1 and 3). Next, it was investigated how these changes resulted in the generation of bioactive peptides and if the abundance of these was affected by lactation month 1 vs. 3. 

## 2. Materials and Methods

### 2.1. Human Milk Samples

HM was collected from six donors in months 1 and 3 of lactation, resulting in a total of 12 HM donor milk samples. All donors delivered vaginally and at term. Information about the donors is presented in Supplementary Table S1, found in the supporting information. The HM samples used in this study are a subset of the samples from participants in the MainHealth cohort [37], registered at clinicaltrials.gov, identification number: NCT05111990. The Central Denmark Region Committees on Health Research Ethics have approved the protocol (J-nr. 1-10-72-296-18). All participants have, before inclusion, signed informed consent and deputy informed consent forms, in accordance with the Declaration of Helsinki II. Before collecting the HM samples, the participants received written instructions about milk collection at home. Participants were asked to avoid sampling the first drops and to manually express the milk into a 40 mL sterile container (Corning, Fisher Scientific, Roskilde, Denmark) around midday and a minimum of two hours after the last breastfeeding. The HM was frozen at −20 °C after collection and kept frozen until further use.

### 2.2. Sample Preparation of the Human Milk Proteome and Peptidome

The proteins in HM were separated into either the proteome representing the intact proteins or the peptidome consisting of the indigenous peptides, according to an earlier published protocol [38], with a few modifications. Then, 200 µL of full milk was aliquoted, 2.5 µL of 1 M 1,4-dithioerythritol (DTE) (D8255-25G from Sigma-Aldrich, Saint Louis, MO, USA) in 20 mM triethylammonium bicarbonate buffer (TEAB) (T7408 from Sigma Aldrich) was added, and the samples were incubated for 30 min. Five µL of 20 mM 2-iodoacetamide (IAA) (8.04744.0100 from MilliporeSigma, Darmstadt, Germany) in 20 mM TEAB was added, followed by incubation in the dark for 20 min. Then, 210 µL of 20% trichloroacetic acid (TCA) (200 mg/mL) (T6339 from Sigma Aldrich) was added to precipitate the intact proteins (the proteome), followed by 10 min of centrifugation at 21,000× *g* at 4 °C. To extract the peptidome, the supernatant was removed and collected in another tube, dried in a vacuum centrifuge and then desalted (Section 2.4). The proteome in the pellet was recovered and dried in the vacuum centrifuge. 

### 2.3. Trypsinization of Intact Proteins in the Proteome

A premixed trypsin/Lys-C mixture (V5111 from Promega, Madison, WI, USA) was used for the digestion of the fraction of intact proteins. Before starting, the trypsin/Lys-C mixture was redissolved in 20 mM TEAB at a concentration of 0.2 µg/µL. The dried pellet from the TCA precipitation was resuspended in 80 µL of 6.5 M urea (29700, Thermo Fisher Scientific, Saint Louis, MO, USA), 20 mM TEAB was added, followed by incubation for 30 min. Then, 17.5 µL of the redissolved trypsin/Lys-C mixture was added, and the HM samples were incubated for 2 h, followed by the addition of 880 µL of 20 mM TEAB. The samples were left for incubation overnight. To stop the digestion, the samples were desalted.

### 2.4. Desalting and Sample Cleaning of Trypsinated Proteome and Peptidome

The trypsinated proteome and the isolated peptidomes from the 12 HM samples were desalted using SPE columns (89851, Thermo Scientific, Rockford, IL, USA), in accordance with the manufacturer’s instructions, with the following modifications: 50% ACN with 0.1% TFA was replaced by 60% ACN with 0.1% TFA and a final elution with 0.1% TFA in ACN was included. The samples were dried in a vacuum centrifuge. To clean the samples of any remaining debris, all the samples were resuspended in 100 µL of 5% ACN with 1% formic acid (FA) (84,865.290, VWR Chemicals, Belgium, Leuven), and filtered through a 10 kDa spin filter (UFC501024, Merck, Ireland, Cork) at 21,000× *g* for 10 min. Here, 20 µL of the proteome digest was added to the filter, and an additional 80 µL of 5% ACN with 1% FA was used to wash the filter; for the peptidome all the sample was used. For both the proteome and peptidome, the filtrate was collected and transferred to a vial for nLC-MS/MS timsTOF Pro II analysis.

### 2.5. Analyzing the Proteome and the Peptidome with the nLC-MS/MS timsTOF Pro II

A nanoElute (Bruker, Solna, Sweden) coupled to a timsTOF Pro II (Bruker, Bremen, Germany) mass spectrometer, with a captive spray ion source was used for the proteomics and peptidomics analysis. The peptides were separated using a Bruker FIFTEEN column (length 15 cm, inner diameter 75 μm, C18 particles with a size of 1.9μm and a pore size of 120 A), with a column temperature of 50 °C. The mobile phases consisted of solvents A (premade water with 0.1% FA, 84,867.320 from VWR chemicals) and B (premade ACN with 0.1% FA, 84,866.320 from VWR chemicals). For the proteome, a 110 min gradient was used, which started at 2% solvent B, then an 80 min linear increase to 25% solvent B, then a 10 min linear increase to 37% solvent B and, lastly, a 10 min linear increase to 90% solvent B, which was held for 10 min. For the peptidome, a 41 min gradient was used, which started at 2% solvent B, then a 22 min linear increase to 17% solvent B, then a 9 min linear increase to 37% solvent B and, lastly, a 1 min linear increase to 95% solvent B, held for 4 min. For all acquisitions, parallel accumulation–serial fragmentation (PASEF) was engaged. All the samples were run in technical duplicates. For analysis of the proteome samples, 1 µL was injected, while for the peptidome samples, 4 µL was injected. Between each sample, a blank sample of solvent A was run to prevent carry-over between the samples. In all cases, the blanks showed minimal to no carry-over from the former sample.

### 2.6. Spectral Data Searching Using FragPipe

Initially, the timsTOF Pro II spectral *.d data were converted to mzML files using ProteoWizard [39], using the following settings: zlib compression was engaged, combined ion mobility scans were engaged, TPP compatibility was engaged, peak picking was engaged on all levels, and scan summing was engaged with a precursor tolerance of 0.05 *m*/*z*, a scan time tolerance of 5 sec and an ion mobility tolerance of 0.1 vs/cm^2^.

FragPipe (Nesvilab, ver. 17.1) [40] was then used for searching the mzML spectral data files against the entire human proteome downloaded from uniport.org (accessed on 17 May 2022). For searching the proteome, the following modifications were made to a default profile: in the MSFagger tab, enzymatic digestion using LysC as the first enzyme and trypsin/P as the second enzyme (to simulate the digestion performed) were used and additional PTMs were enabled (phosphorylation at STYDW, lactosylation and hexosylation both at K), with a maximum of 5 PTMs per peptide. In the validation tab, PeptideProphet with appropriate defaults (search dependent) was chosen instead of Perculator. In the FDR filter and report section the following were enabled: generate peptide-level summary and generate protein-level summary. In the Quant (MS1) tab, IonQuant with match between runs was chosen, with its default settings. For the peptidome, the only difference from the above settings was the choice of enzyme, which was set to non-enzymatic digestion with peptide lengths of 7–50 amino acids (AAs), total PTMs were reduced to 3 per peptide and the split database option was set to 256.

### 2.7. Post-Processing of the FragPipe Data

The data were processed to describe the proteins and peptides in terms of their relative abundance; thus, each identified protein and peptide was calculated as a percentage of the total by calculating the fraction of each protein or peptide of the whole sample as (intensity of the protein or peptide/sum of the intensities of all the proteins or peptides in the sample) ×100. This generated percentage values for the identified proteins and natural peptides, respectively, in the proteome and the peptidome. 

### 2.8. Extracting Proteases, Peptidases and Protease Inhibitors from the Proteome, and Cleavage Sites from the Peptidome

To identify the proteases, peptidases and their inhibitors, the list of identified proteins was analyzed via uniport.org (accessed on 17 May 2022), where the groups of molecular features were used to identify the proteases, peptidases and their inhibitors, respectively. The lists were filtered such that an observation in 2/3 or more of the samples was needed. The lists were further manually inspected and the cleavage motifs for the proteases and peptidases were extracted from MEROPS [23] or the literature. 

To calculate the relative abundance of the observed cleavages giving rise to indigenous peptides, the peptidome data on the peptide relative abundance in percentages were applied. First, the peptides in the peptidome were aligned and summarized such that the peptides were grouped according to their N- or C-terminals, thus peptides with a terminal either side of a cleavage site were grouped, the relative abundance of all the involved peptides were summarized and divided by two. Next, the P4 to P4′ sequence was extracted for each cleavage site from the used FASTA file. This sequence was thus available for later motif matching/searching with the extracted proteases/peptidases motifs.

Finally, to combine the protease data with the peptidome data, the motifs from the observed proteases were used to filter the identified cleavage site motifs. A match was used to assign a protease or peptidase as a possible match to a given cleavage site. Through this procedure, it was possible to convert the peptides in the peptidome to cleavage sites, and assign the proteases and peptidases in the proteome as possibilities to the individual cleavage sites.

To determine the activation indexes for the plasmin and cathepsin D systems, relative amounts of the pro- and/or the activation peptide and the active form were determined. For this, uniprot.org was used to define the borders between these forms. For plasminogen, the activation peptide was found to be between residue positions 20–97, while residues 98–810 were defined as the enzyme. To determine the ratio of enzyme/(enzyme + propeptide), the relative abundance for the two regions, enzyme (AA 98–810) and enzyme + propeptide (AA 20–810), was found by summarizing the peptide relative percentage values for the top three most abundant peptides within each of these regions. In cases where three or less peptides were available, they were all used. For the cathepsin D system, the corresponding calculations were carried out, using positions 21–64 of procathepsin D and positions 21–412 as the enzyme + propeptide.

### 2.9. Bioactive Peptides

Potentially bioactive peptides were identified by searching the obtained list of peptides present in the peptidome against the milk bioactive peptide database (MBPDB), using a similarity of 80% as a criterion [15]. The potential bioactive peptides were grouped according to their bioactivity, and a list of summarized relative abundancies in percentages for the identified bioactivities was generated.

### 2.10. Statistics

For the analysis of the changes in relative protein abundance, linear mixed models in R (ver. 4.1.1.) were applied to the data using the lmerTest package, with the following equation:

Y_lsc_ = µ + L_l_ + S_s_ + (LS)_ls_ + C_c_ + e_lsc_

Here, Y is the dependent variable, μ is the overall mean of the population, L is the fixed effect of lactation, S is the fixed effect of secretor status, C is the random effect of the donor and e is the random residual error, which is assumed to be independent and normally distributed with constant variance.

This approach was applied for both the relative abundance of the proteases, peptidases and their inhibitors, and the relative abundance of the observed cleavage sites, the summed relative abundance of the cleavage sites (to summarize the maximum relative abundance of the cleavage product of the individual proteases and peptidases), and the summed relative abundance of the bioactive peptide groups. In addition, for an observation to be relevant, the involved protein, protease, peptidases or inhibitors should be observed in at least 2/3 of all the samples. 

For the analysis of the relative protein composition with the proteome and peptidome, the calculated relative abundance in percentages was used as input data. For the analysis of the cleavage sites, the calculated relative abundance of the cleavage sites in percentages was used for the analysis. For the analysis of the bioactive groups, the calculated relative abundance of the group of bioactive peptides in percentages was used for the analysis.

## 3. Results

In the HM proteomes, the number of identified proteins per sample varied from 1324 to 2772 proteins and covered a total of 4365 different proteins, with an average of 1903 ± 445 proteins at lactation month 1 and 1775 ± 328 at lactation month 3; no statistically significant differences in the protein counts between lactation months 1 and 3 were found (Table 1). In the peptidome, the number of identified peptides per donor varied from 208 to 5039, covering a total of 13,158 unique peptides, with an average of 3577 ± 1006 peptides at lactation month 1 and 2575 ± 1358 peptides at lactation month 3 (Table 1). There were no significant differences in the peptide count on indigenous peptides between lactation months 1 and 3. The indigenous peptides in the peptidome were found to originate from 60–445 different proteins corresponding to 2.8–22.6% of unique proteins per sample (Table 1). Not all the identified proteins or peptides were observed in all the samples, thus explaining the large variation in the observed protein and peptide counts between individual donors (Table 1).

### 3.1. Most Abundant Human Milk Proteins in the Proteome and Peptidome

**Figure 1:**
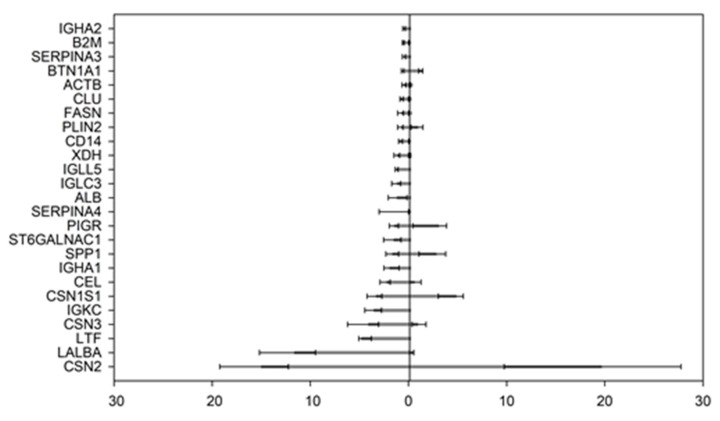
The top 25 most abundant proteins identified in the proteome. Bars to the left represent the relative abundance of the protein in the proteome, while the bars to the right represent the relative abundance of the protein in the peptidome. Relative abundancies (in% of total protein) are displayed on the *x*-axis with bar plots, where the bar represents the median relative abundance and the inner and outer whiskers represent the 1st and 3rd quartiles, respectively. The *y*-axis represents the gene names for the identified proteins according to uniprot.org.

The top 25 most abundant proteins in the HM samples are ranked according to their abundance in the proteome (Figure 1). The sum of the top 25 most abundant proteins constituted 63.2 ± 3.6% of the total protein in the proteome. In the proteome (Figure 1), the most abundant proteins were β-CN (CSN2), α-LA (LALBA), lactoferrin (LTF), κ-CN (CSN3), immunoglobulin kappa constant chain (IGKC), α_S1_-CN (CSN1S1), bile salt-activated lipase (CEL), immunoglobulin heavy constant α-1 chain (IGHA1) and osteopontin (SPP1). Similarly, the most abundant proteins in the peptidome were β-CN, α_S1_-CN, titin (TTN), collagen α-1(XVII) chain (COL17A), polymeric immunoglobulin receptor (PIGR), mediator of RNA polymerase II transcription subunit 19 (MED19), osteopontin, perilipin-2 (PLIN2) and macrophage mannose receptor 1 (MRC1) (not shown). In contrast with the proteome, α-LA was only the 10th most abundant protein in the peptidome, and LTF was not detected in the peptidome. The differences between the proteome and peptidome could indicate preferential hydrolysis of some HM proteins over others.

Among the top 25 most abundant proteins in the proteome (Figure 1), both the caseins (α_S1_-, β- and κ-CN) and the major whey proteins (α-LA, LTF and SPP1) were identified. The relative abundance of the group of caseins was 22.9 ± 6.7% and 25.4 ± 12.5% of the total relative abundance within the proteome and peptidome, respectively. The relative abundance of the group of major whey proteins were 17.3 ± 5.3% and 4.4 ± 4.3% within the proteome and peptidome, respectively. This could indicate a preference for hydrolyzing the casein proteins in HM over the major whey proteins in HM. 

### 3.2. Proteases Identified and Quantified in the Human Milk Proteome

The list of total identified proteins in the HM samples was uploaded to uniprot.org and searched for molecular features. A total of 143 proteins were identified as having proteolytic activity. Of these, 39 were present in at least 2/3 of the samples (Table 2). Among the 39, 15 were categorized as peptidases, including both amino-, carboxy- and di-peptidases. Thus, of the 39 proteases, 24 were known proteinases, 10 were aminopeptidases, seven were carboxypeptidases and two were dipeptidases. Furthermore, some of the proteases, such as CTSB, were known from several classes (Table 2). Plasminogen (PLG, covering both plasminogen and plasmin) was identified at some level in all the HM samples. Three different cathepsins, in order of abundance, cathepsin D (CTSD), cathepsin B (CTSB) and cathepsin S (CTSS), were all identified in the HM proteome. Interestingly, according to the presented study (Table 2), cathepsin D was more abundant than plasmin/plasminogen. Other proteases identified in at least 2/3 of the samples include caspases 3 and 6 (CASP3, CASP6), legumain (LGMN) and kallikreins, including kallikrein-6, -8 and plasma kallikrein.

### 3.3. Using the Relative Abundance of Peptides to Calculate the Activation Degree of Proteases

**Figure 2:**
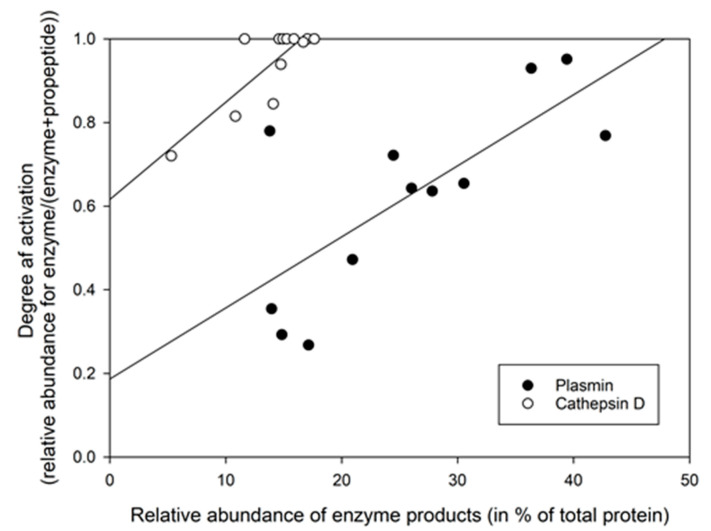
Relationship between the degree of activation (*y*-axis) for plasmin and cathepsin D enzyme systems and relative abundance of cleavage products (peptides in peptidome) ascribed to either enzyme system based on cleavage specificities. The *x*-axis indicates the relative abundance of the enzyme products, i.e., peptides in the peptidome. The *y*-axis shows the activation degree (enzyme/(enzyme + proenzyme)) for plasminogen and procathepsin D, based on the relative abundance of peptides originating from the activation peptide regions and peptides from the mature enzyme.

Using the relative abundance of peptides representing either zymogen/propeptides or the active enzyme (representing the proteases in both zymogen and active forms), an index of activation degree was calculated for the two most well-known HM proteases. Plasmin/plasminogen and cathepsin D/procathepsin D, also present as some of the most abundant proteases in the proteome (Table 2) and are shown relative to the amount of cleavage products (Figure 2). The ratio of enzyme/(enzyme + propeptide) would thus indicate the level of activation of the proenzymes, where a value close to 1 indicates full activation (Figure 2).

From our data, the level of activation of the cathepsin D system was observed to be higher than for the plasmin system (Figure 2). It was further found that the level of activation for plasminogen was significantly higher in month 3 (0.75 ± 0.10) compared to month 1 (0.49 ± 0.23) (*p* = 0.0368), while for procathepsin D no significant difference was found between the two lactation stages. Further, in Figure 2, it can be seen that for both plasmin and cathepsin D, there is a relationship between the activation degree and the level of ascribed cleavage products.

### 3.4. Determining Protease Activity Based on the Relative Abundance of Their Ascribed Cleavage Products

**Figure 3:**
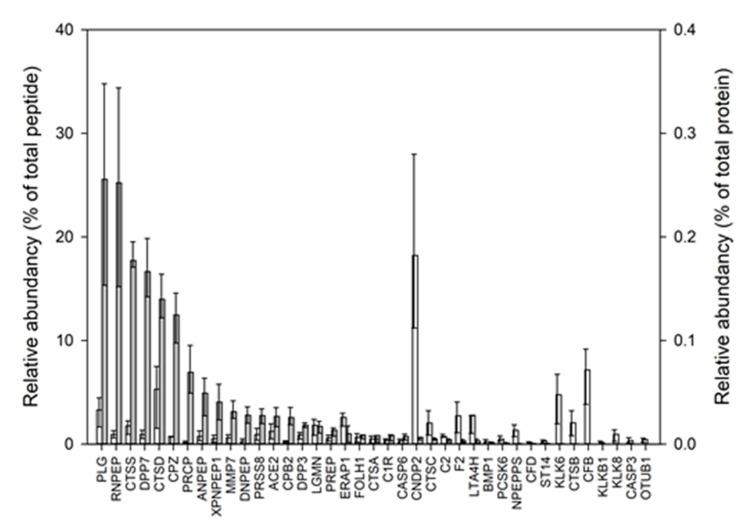
Average relative abundance (*y*-axis) of the 39 proteases (*x*-axis) identified in the proteome (white bars, right *y*-axis) or giving rise to peptides found in the peptidome, based on enzyme specificities (grey bars, right *y*-axis), and as represented in at least 2/3 of the HM samples. The top and bottom whiskers represent the 1st and 3rd quartiles, respectively.

Based on the summarized relative abundance of the peptides in the peptidomes matched to MEROPS extrapolated cleavage motifs in the proteases (Table 2), the activity of the 39 proteases in the HM was calculated as the maximum relative abundance by summarizing the abundance of each cleavage site assigned to it, these were correlated to the relative abundance of the proteases (Figure 3). Furthermore, the relative abundance of each of the proteases in the proteome (Figure 3, second *y*-axis) showed that the top 10 proteases with the highest relative abundance of cleavage products were: plasmin (PLG), aminopeptidase B (RNPEP), cathepsin S (CTSS), dipeptidyl peptidase 2 (DPP7), cathepsin D (CTSD), carboxypeptidase Z (CPZ), lysosomal Pro-X carboxypeptidase (PRCP), aminopeptidase N (ANPEP), Xaa-Pro aminopeptidase 1 (XPNPEP1) and matrilysin (MMP7). Among these, four were proteases (PLG, CTSS, CTSD and MMP7), and the relative abundance of their cleavage products was found to be 25.6 ± 9.6, 17.7 ± 3.5, 13.6 ± 3.1 and 3.1 ± 1.3% for PLG, CTSS, CTSD and MMP7, respectively. In terms of possible cleavage sites per sample, this would translate to an average of 509.91 ± 204.95, 414.58 ± 54.95, 329.91 ± 137.94 and 33.83 ± 13.10 sites for PLG, CTSS, CTSD and MMP7, respectively. Together these four proteases had the potential to account for up to 60.0 ± 9.0% of the relative abundance of the peptidome, corresponding to 1288.25 ± 504.02 individual cleavage sites per sample. This indicates significant contributions from classic milk proteases, such as plasmin and cathepsin D, with significant contributions from other proteases.

### 3.5. Protease Inhibitors Identified and Quantified in the Human Milk Proteome

A total of 26 protease inhibitors were present in 2/3 of the samples and are, according to uniprot.org, known to inhibit proteases and peptidases (Table 3). For each protease inhibitor, the target class of protease is indicated (Table 3). Twenty of these were serine protease inhibitors (serpins), four were thiol protease inhibitors, and two were metalloprotease inhibitors. In addition, α2-macroglobulin (A2M) was observed. A2M is the only observed inhibitor that can inhibit multiple classes of proteases, namely serine, thiol, and aspartic proteases, and were thus the only inhibitor able to inhibit aspartic proteases, like cathepsin D. Plasminogen can be inhibited by LTF [27,33] and kininogen-1 (KNG1), Protein AMBP (AMBP), amyloid-β precursor-like protein 2 (APLP2) and three serpins (SERPINA1, SERPINF2 and SERPINI1) were found. Apart from α2-macroglobulin, for the thiol class of cathepsins, cathepsin B and S can potentially be inhibited by the thiol inhibitors (cystatin-C, kininogen-1, cystatin-B (CSTB), WAP four-disulfide core domain protein 2 (WFDC2), calpastatin (CASB) and α2-macroglobulin) (Table 3).

### 3.6. Preferred Cleavage Sites in Human Milk Proteins by the Indigenous Proteases

From the 13,158 unique peptides in the peptidome (Table 1), 9280 cleavage sites were identified, excluding those at the protein terminals. Of these, the average sample had 2101.75 ± 814.88 cleavage sites. The motifs in the 39 proteases and peptidases as found in 2/3 of the samples, could cover 7536 of the 9280 cleavage sites. Thus, 1711.41 ± 657.30 of the 2101.75 ± 814.88 cleavage sites per sample were covered by these 39 proteases and peptidases, which was equal to 79.6 ± 2.3% or the relative abundance of the peptidome in each sample.

**Figure 4:**
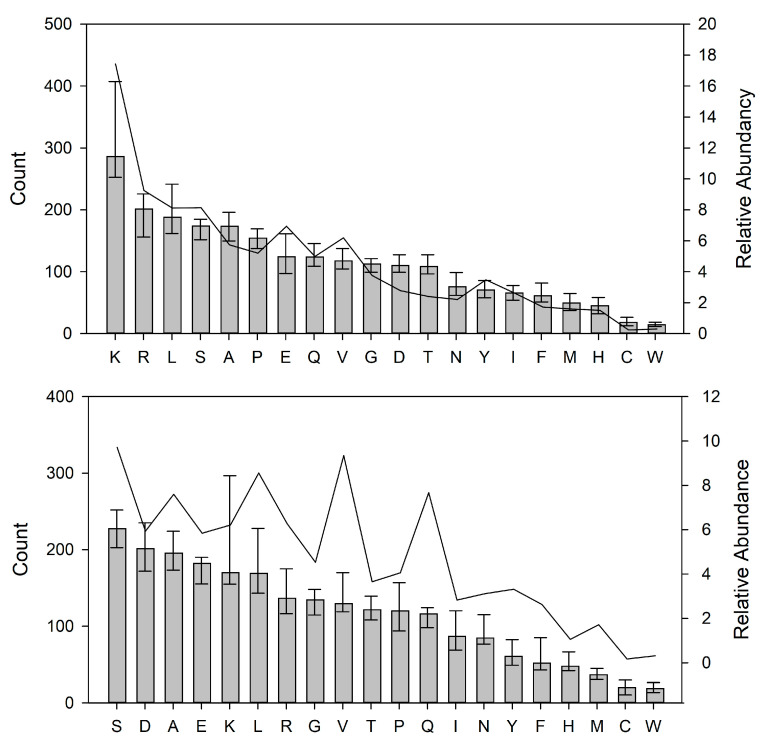
Residues at P1 (**top panel**) and P1′ (**bottom panel**) positions of the peptides in the peptidome of the indigenous peptides. The amino acid residues are represented by one-letter codes on the *x*-axis. The bars represent the median count of the total observations within each of the samples. The top and bottom whiskers represent the 1st and 3rd quartiles, respectively. The line represents the average summarized peptide intensities representing each residue across each sample. The left *y*-axis shows the peptide counts; the right *y*-axis shows the peptide intensities.

To investigate the preference for cleavage sites in the HM proteins for the 39 identified indigenous proteases in Table 2, the P1 and P1′ positions of the cleavage sites in the peptidome were mapped; both the raw counts on the observations and the stacked intensities for the individual amino acid residues are shown (Figure 4). From these, it is evident that the dominating P1 residues were K > R > L > S, with K being both the most frequent and most abundant residue at the P1 position. The P1′ position was dominated by S > D > A > E, with S being the most frequent and most abundant residue at the P1′ position. 

#### Enzyme Activity on the Caseins and Whey Proteins

**Figure 5:**
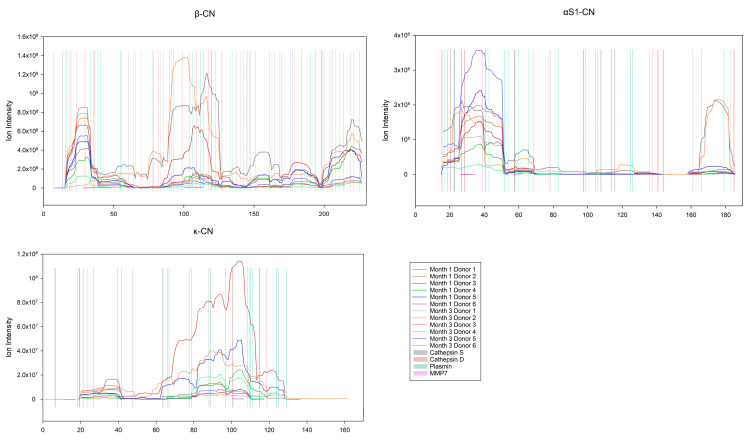
Shows a peptide intensity map of the caseins in the peptidome (graphs) with cleavage sites for the four most active proteases (vertical lines). The *x*-axis shows the amino acid position, including signal peptides. The *y*-axis shows the ion intensity (does not apply to the vertical lines). The summarized intensities were calculated using PepEx and plotted as graphs for the individual donors (lines). The color code applies to all three plots.

**Figure 6:**
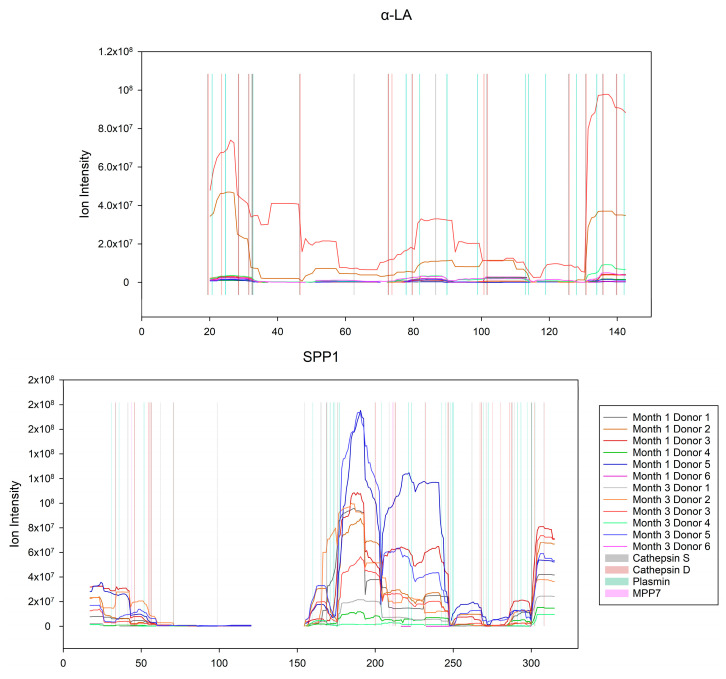
Shows a peptide intensity map of the caseins in the peptidome (graphs) with cleavage sites for the four most active proteases (vertical lines). The *x*-axis shows the amino acid position, including signal peptides. The *y*-axis shows the ion intensity (does not apply to the vertical lines). The summarized intensities were calculated using PepEx and plotted as graphs for the individual donors (lines). The color code applies to both plots.

To investigate the effect of the top four most active proteases, PLG, CTSS, CTSD and MMP7, on the caseins (α_S1_-, β- and κ-CN) and major whey proteins (α-LA and SPP1, note that LTF was not observed in the peptidome), PepEx [41] was used to generate peptide maps using the peptide intensities, and not the relative abundance of the peptides. The generated maps were overlaid with the possible cleavage sites for the four proteases (Figure 5 and Figure 6). Interestingly, out of the 13,158 unique peptides in the peptidome, 3472 could be assigned to the group of caseins, of which 2103 peptides were from β-CN. Out of the 9280 observed cleavage sites, 816 were found on the caseins (483) and major whey proteins (333), of these 816 cleavage sites 366 could be explained by the four most active proteases, PLG, CTSS, CTSD and MMP7. Of the 483 cleavage sites on the caseins, 191 cleavage sites could be explained by the four most active proteases. On β-CN alone, a total of 216 cleavage sites were observed, which, considering that the length of human β-CN is 226 amino acids, is a lot. Of the 216 cleavage sites on β-CN, 105 could be explained by the four most active proteases. From this, it is clear that a small number of proteases have the potential to generate a large portion of the observed peptidome.

### 3.7. Bioactive Peptides

**Figure 7:**
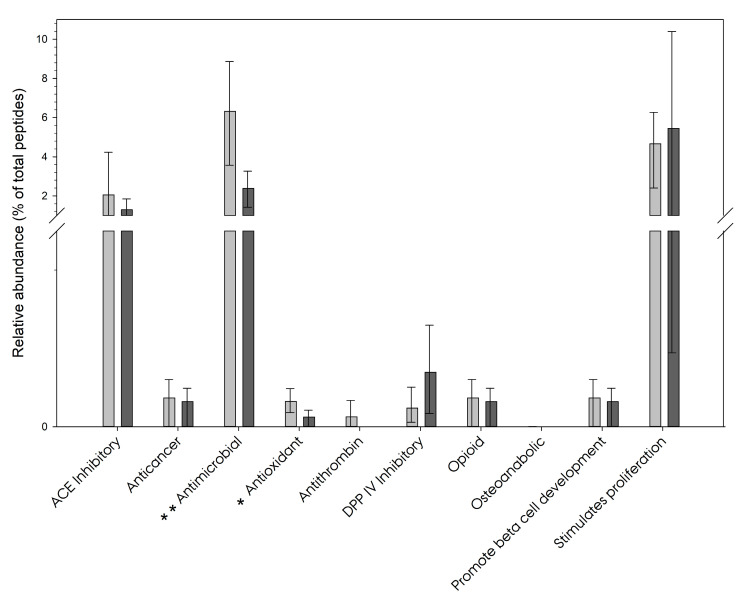
Shows the relative abundance of the groups of bioactive peptides in the peptidome. Light grey bars represent lactation month 1, while dark grey bars represent lactation month 3. The bar represents the average, while the top and bottom whiskers represent the 1st and 3rd quartiles, respectively. Of the ten groups of bioactive peptides, the relative abundances of the antimicrobial and antioxidant groups were found to be significantly different between lactation months 1 and 3. The break in the *y*-axis is from 0.25 to 2. Asterisks denote the level of significance between the lactation groups: *, *p* ≤ 0.05; **, *p* ≤ 0.01.

The peptides in the peptidome were searched for known bioactive peptides in the milk bioactive peptide database (MBPDB) [15]. A similarity score of 80% was used. Of the 13,158 peptides in the peptidome, 282 potentially bioactive peptides were identified, representing a possibility of ten different categories of bioactivity. When calculating the relative abundance in the peptidome of the potentially bioactive peptides, the sum of these was found to be 11.2 ± 4.3% of the total abundance in the HM peptidome (Figure 7). Considering the relative abundance, the largest group of bioactive peptides were antimicrobial peptides. The relative abundance of this group within the peptidome was significantly higher in lactation month 1 compared with lactation month 3 (*p* = 0.006). 

The 103 involved peptides were all derived from the C-terminus of β-CN (Figure 5). Furthermore, the relative abundance of the group of antioxidant peptides was found to be significantly higher in lactation month 1 compared to month 3 (*p* = 0.021).

## 4. Discussion

In the present study, the proteomes and the peptidomes in six HM donor samples at lactation months 1 and 3 were profiled using nLC-MS/MS timsTOF Pro II, and based on the peptide and protein intensities, the relative abundance of the HM peptidomes and proteomes were calculated. The sensitivity of this system enabled detailed profiling not only of the indigenous peptides, but also direct determination of the abundance of the present proteases, their inhibitors and the resulting cleavage sites within the major HM proteins, thus allowing for extensive mapping of pre-digestion and the released bioactive peptides.

### 4.1. Identification and Abundance of Proteins and Peptides in the HM Proteome and Peptidome

In the proteome of HM, the average number of identified proteins was 1903 and 1775 in months 1 and 3 of lactation, respectively. Similarly, in the peptidome, an average of 3577 and 2575 indigenous peptides were identified in months 1 and 3 of lactation, respectively. In all cases, the donor variation was large resulting in no significance in the peptide or protein counts between months 1 and 3 (Table 1). 

The obtained relative abundance indicated that the major proteins in the analyzed HM proteomes were: β-CN, α-LA, LTF, κ-CN, IGKC, α_S1_-CN, CEL, IGHA1 and SPP1 (in declining order) (Figure 1). In comparison, the ranking reported by for e.g., Zhu et al. (2021) [2] was only slightly different: β-CN, α-LA, LTF, κ-CN, α_S1_-CN, serum albumin, immunoglobulin α-1, CEL and polymeric immunoglobulin receptor (in declining order), showing many similarities and only minor differences in the order of the main HM proteins, which may be caused by differences in methodologies or variations between donor milk samples from individual mothers. For the peptidome, in the present study, the ranking of the five most abundant proteins giving rise to the indigenous peptides was: β-CN, α_S1_-CN, titin, collagen α-1(XVII) chain and polymeric immunoglobulin receptor (not shown, in declining order). In comparison, Zhu et al. [2] obtained a slightly different order, namely β-CN, SPP1, α_S1_-CN, polymeric immunoglobulin receptor and κ-CN (in declining order), showing selective hydrolysis of the casein proteins (Figure 1). This shows that the caseins are preferentially hydrolyzed over the major human whey proteins α-LA, SPP1 and LTF, thus validating earlier studies [11]. The predominant presence of PIGR in both the proteome and peptidome complies with earlier studies [2,11,42]. This finding represents the role of the secreted form of the PIGR protein, referred to as the secretory component, being the hydrolysis product of PIGR during transport of secretory immunoglobulin A from the basolateral to the apical side of the secretory cells [43,44]. 

### 4.2. Abundancies of Different Protease Systems in Human Milk

A total of 39 proteases, including 15 categorized as peptidases, were identified in at least 2/3 of the HM samples (Table 2). Among these, plasminogen, cathepsin D and kallikrein are among the more well-known proteases present in milk [18,35]. Apart from these, cathepsins B and S were also identified and quantified directly in the proteome. These have been detected earlier in milk, either based on immunological detection [16,35] or via ascribed peptides in the peptidome [16,35]. A somewhat surprising observation is that, in the present study, the amount of cathepsin D as determined based on its abundance in the proteome, was higher than that of plasminogen. Normally, plasminogen is reported as the major protease in milk [17,18], but for e.g., donor variation and differences in the cell counts may affect the levels of cathepsins. Furthermore, as mentioned, many of the reported levels of milk proteases are based on activity assays [17,18], which may generate results that are different from LC-MS/MS-based results that are focused on the peptide and protein abundance.

Further, comparing the number of observed proteases and peptidases in the present study with those of earlier LC-MS/MS-based studies, such as Dallas et al. [16], Nielsen et al. [12] and Zhu et al. [2], it is interesting to note that here a larger number of enzymes were quantified. These differences could be explained by differences in the sample preparation and purification techniques and differences in the sensitivity of the used LC-MS/MS systems; the present study made use of timsTOF Pro II, whereas Nielsen et al. [12] used an Orbitrap Fusion Lumos instrument, Dallas et al. [16] used a Q-TOF instrument and Zhu et al. [2] used a Q-Exactive Plus Orbitrap system. Nielsen et al. [12] reported the order of abundance for the produced indigenous peptides to indicate that the order of activity among the proteases was: plasmin, elastase, thrombin and kallikrein-6. Of the 39 reported proteases in the present study, not all have previously been reported in milk, including HM, for e.g., caspases 3 and 6 (CASP3, CASP6), metalloprotease (MMP7), kallikreins 6, 8 and plasma kallikrein (KLK6, KLK8 and KLKB1), as well as a range of different types of peptidases, including both amino- and carboxypeptidases, which are involved in N- and C-terminal trimming of the generated peptides [17]. New, additional knowledge reported in the present study involves the reporting on the quantitative levels for a larger list of proteinases and peptidases compared with earlier studies. 

A new approach was taken for estimating the degree of protease activation by estimating the balance between the proenzyme and active protease using the ratio of peptide abundancies representing the proenzyme (and/or the activation peptides) for both the zymogens and the active enzymes. This ratio was used to determine the level of activation of the protease systems for plasmin and cathepsin D. Initially, calculating these ratios for the four most active proteases was attempted (Figure 2 and Figure 3), representing different enzyme classes in the HM, namely plasmin, cathepsin D, cathepsin S and matrix metalloprotease 7 (Table 2, Figure 3). However, it was only possible to perform the calculations for plasmin and cathepsin D. The degree of activation of plasminogen was found to be significantly higher in lactation month 3 compared to month 1, and this was shown to be reflected in the relative amount of potential degradation products generated by plasmin products as observed in the peptidome. Even though the trend was not perfect, it was observed that there is a relationship between the degree of activation and the maximum relative abundance of products ascribable to plasmin and cathepsin D activities in the peptidome. 

For cathepsin D, the protein coverage in the proteome data was not sufficient to detect the peptides representing the propeptide sequence from procathepsin D in all samples, and in these cases the activation index came out as 1 (Figure 3); however, from the alignment of the activation (proteome) and the maximum relative abundance of the cleavage products (peptidome), it was observed that the samples with this issue, were also among those with more product. Thus, also for cathepsin D, it appears that the degree of activation can provide some explanation for the observed level of the product. For both the plasmin and cathepsin D systems in HM, it should be noted that the used approach would include all possible peptides in the peptidome to calculate the total relative abundance of degradation products ascribed to these two protease systems, thus some cleavage sites can be shared with other proteases. In either case, it is interesting to observe that there seems to be a relationship between the degree of activation and the total relative abundance of the protease products. For cathepsin D, it could be speculated that the high activity could be connected to the few inhibitors that are able to inhibit it (Table 2 and Table 3).

### 4.3. Abundance of Different Protease Inhibitors in Human Milk

A total of 26 protease inhibitors, also called antiproteases, were identified in the HM proteome. This is a higher number than earlier reported for HM, where especially serpins were reported [18]. Previously, using proteomic methods and/or by using immunoassays, α_1_-antichymotrypsin, α_1_-antitrypsin, antithrombin III, α_2_-macroglobulin, α_2_-antiplasmin and plasma serine protease inhibitor have been reported, thus including here further protease inhibitor classes than the most well-described HM serpins. As an example of an inhibitor of cysteine proteases, calpains are inhibited by calpastatin (CAST), which was observed in the proteome of HM. Mammalian inhibitors of aspartic proteases, like cathepsin D, are rare, but α_2_-macroglobulin (A2M) is a broad inhibitor, blocking the interaction between proteases and their substrates [45,46]. Moreover, α_2_-Macroglobulin was found in 11 samples of HM, with an average relative abundance of 0.03 ± 0.01% in the proteome; in addition, α_2_-Macroglobulin was the only aspartic protease inhibitor found in the present study.

### 4.4. Selective Hydrolysis and Preferred Cleavage Site Motifs in Human Milk

On average, 3577 and 2575 unique peptides were identified as indigenous peptides in the peptidome at months 1 and 3, respectively (Table 1). Despite the high numbers of indigenous peptides in the present study, these only corresponded to an average of 326 and 306 different proteins at months 1 and 3 of lactation, respectively. This again corresponded to an average of 16.4 ± 4.2% and 13.4 ± 6.2% of the total count of unique proteins (from both the peptidome and proteome) for months 1 and 3 of lactation, respectively. This shows that the degradation processes in the HM system are highly selective, as reported in earlier studies [11]. In three comparable studies, 1107 (Nielsen et al. [12]), 445 (Dallas et al. [16]) and 2096 (Zhu et al. [2]) unique peptides were observed in the peptidomes, these differences could be ascribed to donor variation, as well as instrumental differences. 

The protein profiles of the peptidome and proteome are quite different (Section 3.1), this is also reflected in the peptide pools of the former. When focusing on HM casein (α_S1_-, β- and κ-CN) and major whey proteins (α-LA, LTF and SPP1), it is interesting to observe that no peptides were identified from LTF in the peptidome. It was further confirmed that indigenous peptides in the peptidome originated from specific parts of the protein sequences, as shown for β-, α_S1_- and κ-CN (Figure 5), as well as for the whey proteins α-LA and SPP1 (Figure 6). Further, it is evident that the summarized proportion of caseins and major whey proteins are differently represented between the proteomes and peptidomes (Section 3.1). While the summarized relative abundance of the caseins in the proteome and the peptidome were found to be comparable, the relative abundance of the major whey proteins were found to differ between the proteome and peptidome. The relative abundance of the major whey proteins constituted a smaller portion of the peptidome as compared with that of caseins. This indicated that the caseins are favored for proteolysis, while the major whey proteins are more resistant. 

When comparing the relative abundance of the individual proteins in further detail (Figure 1), it was found that both β- and α_S1_-CN make up larger relative proportions of the peptidome (19.22 ± 11.9% and 5.05 ± 4.07%, respectively) than the proteome (14.59 ± 5.04% and 3.37 ± 0.80%, respectively). In contrast, κ-CN and α-LA make up a larger portion of the proteome (4.36 ± 1.89% and 11.93 ± 3.11%, respectively) than the peptidome (1.13 ± 1.06% and 1.83 ± 3.88%, respectively). LTF was observed in the proteome only (4.70 ± 1.25%), while SPP1 was found to make up a larger portion of the peptidome (2.54 ± 1.55%) compared to the proteome (1.56 ± 0.66%). This could indicate that the whey proteins, to some extent, resist digestion by the naturally present proteases in HM. 

Among the more obvious examples of exopeptidase activities are the ones on the N- and C-terminals of β-CN (Figure 5), generating “ladders” of peptides [12,16], where one of the terminals is being trimmed. One of the more interesting cases is found in β-CN at residues 33–40 (Figure 5), where a sharp increase in peptide intensity is observed, which may be explained by a combination of two milk proteases, plasmin and carboxypeptidase Z, as many Lys residues are found in the area at positions 33, 35, 38 and 40. This would allow plasmin to cleave at these positions, thus placing the Lys residues at the C-terminus of the new peptides, where Lys is then available for carboxypeptidase Z. Further, by matching the observed cleavage sites to MEROPS, uniprot.org cleavage motifs, the κ-CN 106|107 position was found to match an elastase cleavage, while the α-LA 130|131 position was ascribed to cathepsin S and SPP1 203|204 to plasmin [47]. 

### 4.5. Bioactive Peptides

The 13,158 identified peptides in the peptidome were searched against the MBPDP by Nielsen et al. [15]. In total, 282 bioactive peptides were identified with 402 different bioactivities, thus some were either known or 80%, or more, similar to multiple different bioactivities. An example of such a peptide, is that of 168-LWSVPQPK-175 in β-CN has been identified with both ACE-inhibiting and antioxidant activities [48]. 

On average, these bioactive peptides were found to make up 11.24 ± 4.34% of the peptidome. Considering that the average peptidome was observed with 3076.00 ± 1295.96 peptides, and that 284 is less than 1/10 of this, it is interesting to observe that they make up a larger portion of the relative abundance. This also indicates that these peptides are likely to be among the peptides with a higher-than-average relative abundance which, considering that they are functional, makes sense. This dilemma does allow one to wonder if the generation of the peptidome is truly random or if the process somehow is controlled, maybe to provide extra functionality to HM in the form of bioactive peptides. Exploring this line of thinking would require additional studies.

## 5. Conclusions

Thirty-nine different proteases, including a range of peptidases and 26 protease inhibitors were observed in human milk, with cathepsin D being the most abundant among the proteases in the proteome. However, based on the peptidome, the protease giving rise to most peptides was found to be plasmin. This may reflect that the pH of milk favors plasmin activity. Using specific peptides, representing activation peptides and peptides from active enzyme regions, it was possible to use these MS-based abundances to calculate the activation indexes of the plasmin and cathepsin D systems. It was found that the activation degree of the procathepsin D system was higher than for plasminogen, but also that the activation degree correlated with the level of indigenous peptide products in the peptidome. Furthermore, the activation level of plasminogen was higher in lactation month 3 than in month 1. Despite the large variation between donors, a high number of indigenous peptides were detected in most HM samples, confirming the pre-digestion of especially the caseins in HM. The peptides in the peptidome were searched for similarities to known bioactive peptides, and 282 peptides were identified to cover 10 different bioactive functionalities, where the group of antimicrobial peptides were found to be up-regulated at month 1 of lactation compared to month 3.

## Figures and Tables

**Table 1:** The number of identified proteins and peptides in the proteome and the peptidome in the donor milk samples (n = 6) at lactation months 1 and 3. For both the proteins and peptides, the number of unique entries across the proteome and peptidome is described together with the overlapping entries observed between the proteome and peptidome as raw count and percentage (%) of the unique entries. For all of these, the average and the standard deviation are found across all donors within each lactation stage. The *p*-value column shows the calculated *p*-value for investigating the significance of the raw values when comparing values from months 1 and 3 with a double-sided *t*-test with different variances.

Time	Month 1							Month 3							*p*-Value
Donor	1	2	3	4	5	6	Average	1	2	3	4	5	6	Average	
**Proteins identified**															
Proteome	1996	1697	1350	2001	1601	2772	1903 ± 445	2137	1755	1550	1324	1623	2262	1775 ± 328	0.62
Peptidome	302	445	307	292	315	294	326 ± 54	60	306	351	319	274	306	269 ± 96	0.29
Unique proteins ^1^	2132	1973	1516	2160	1771	2889	2074 ± 426	2164	1904	1761	1486	1750	2390	1909 ± 295	0.50
Overlap ^2^	166	169	141	133	145	177	155 ± 16	33	157	140	157	147	178	135 ± 47	0.41
Overlap in% of unique ^3^	7.79	8.57	9.30	6.16	8.19	6.13	7.69 ± 1.18	1.52	8.25	7.95	10.57	8.40	7.45	7.36 ± 2.79	0.81
Peptidome in% of unique ^4^	14.17	22.55	20.25	13.52	17.79	10.18	16.41 ± 4.22	2.77	16.07	19.93	21.47	15.66	12.80	13.35 ± 6.20	0.64
**Peptides identified**															
Proteome	13,065	10,424	6848	12,490	8978	18,396	11,700 ± 3649	14,127	9693	8808	7775	11,036	16,255	11,282 ± 2995	0.85
Peptidome	2464	5039	2603	4295	2747	4316	3577 ± 1006	208	2560	4627	2868	1830	3355	2575 ± 1358	0.22
Unique peptides ^1^	15,479	15,402	9380	16,719	11,687	22,640	15,218 ± 4163	14,334	12,164	13,403	10,553	12,828	19,525	13,801 ± 2810	0.54
Overlap ^2^	60	72	82	75	45	80	69 ± 13	7	100	39	99	44	95	64 ± 36	0.78
Overlap in% of unique ^3^	0.39	0.47	0.87	0.45	0.39	0.35	0.49 ± 0.18	0.05	0.82	0.29	0.94	0.34	0.49	0.49 ± 0.31	0.99
Peptidome in% of unique ^4^	15.92	32.72	27.75	25.69	23.50	19.06	24.11 ± 5.52	1.45	21.05	34.52	27.18	14.27	17.18	19.92 ± 10.67	0.39

^1^ = Unique peptides/proteins is a list of entries where each entry can only be present once even if it is found in both the proteome and peptidome. ^2^ = Overlapping peptides/proteins are peptides/proteins that are found in both the proteome and peptidome. ^3^ = Overlap in percentage of unique is the overlapping entries calculated as a percentage of the number of unique entries. ^4^ = Peptidome in percentage of unique is the peptidome as a percentage of the number of unique entries.

**Table 2:** List of the 39 proteases identified in at least 2/3 of the HM samples. Average ± St.dev. indicates average relative abundance +/− standard deviation in% of total protein. The order of the proteases is sorted from most to least abundant, based on the average of the relative abundance.

Protein ID ^1^	Gene Name	Protease	Average ± St.dev ^2^ (%)	Type ^1^	Class ^1^
Q96KP4	CNDP2	Cytosolic non-specific dipeptidase	0.18 ± 0.09	D	M
P00751	CFB	Complement factor B	0.07 ± 0.05	P	S
P07339	CTSD	Cathepsin D	0.05 ± 0.07	P	A
Q92876	KLK6	Kallikrein-6	0.05 ± 0.03	P	S
P00747	PLG	Plasminogen	0.03 ± 0.02	P	S
P09960	LTA4H	Leukotriene	0.03 ± 0.02	A	M
P00734	F2	Prothrombin	0.03 ± 0.02	P	S
Q9NZ08	ERAP1	Endoplasmic reticulum aminopeptidase 1	0.03 ± 0.02	A	M
P07858	CTSB	Cathepsin B	0.02 ± 0.02	C, D, P ^3^	T
P53634	CTSC	Dipeptidyl peptidase 1	0.02 ± 0.01	A	T
Q99538	LGMN	Legumain	0.02 ± 0.01	P	T
P25774	CTSS	Cathepsin S	0.02 ± 0.01	P	T
P55786	NPEPPS	Puromycin-sensitive aminopeptidase	0.01 ± 0.01	A	M
Q9BYF1	ACE2	Angiotensin-converting enzyme 2	0.01 ± 0.01	C, P	M
O60259	KLK8	Kallikrein-8	0.01 ± 0.01	P	S
Q16651	PRSS8	Prostasin	0.01 ± 0.01	P	S
Q9H4A4	RNPEP	Aminopeptidase B	0.01 ± 4.47 × 10^−3^	A	M
Q9UHL4	DPP7	Dipeptidyl peptidase 2	0.01 ± 0.01	A	S
Q9NY33	DPP3	Dipeptidyl peptidase 3	0.01 ± 0.01	A	M
P15144	ANPEP	Aminopeptidase N	0.01 ± 0.01	A	M
P06681	C2	Complement C2	0.01 ± 0.01	P	S
Q66K79	CPZ	Carboxypeptidase Z	0.01 ± 0.01	C	M
Q04609	FOLH1	Glutamate carboxypeptidase 2	0.01 ± 4.23 × 10^−3^	C	M
P09237	MMP7	Matrilysin	0.01 ± 0.01	P	M
P48147	PREP	Prolyl endopeptidase	0.01 ± 0.01	P	S
Q9NQW7	XPNPEP1	Xaa-Pro aminopeptidase 1	0.01 ± 3.57 × 10^−3^	A	M
P10619	CTSA	Lysosomal protective protein	0.01 ± 4.56 × 10^−3^	C, P	S
P29122	PCSK6	Proprotein convertase subtilisin/kexin type 6	<0.01	P	S
Q96FW1	OTUB1	Ubiquitin thioesterase OTUB1	<0.01	P	T
P00736	C1R	Complement C1r subcomponent	<0.01	P	S
P42574	CASP3	Caspase-3	<0.01	P	T
Q9Y5Y6	ST14	Suppressor of tumorigenicity 14 protein	<0.01	P	S
P55212	CASP6	Caspase-6	<0.01	P	T
Q9ULA0	DNPEP	Aspartyl aminopeptidase	<0.01	A	M
P13497	BMP1	Bone morphogenetic protein 1	<0.01	P	M
Q96IY4	CPB2	Carboxypeptidase B2	<0.01	C	M
P03952	KLKB1	Plasma kallikrein	<0.01	P	S
P42785	PRCP	Lysosomal Pro-X carboxypeptidase	<0.01	C	S
P00746	CFD	Complement factor D	<0.01	P	S

^1^ According to uniprot.org. Type indicates the protease type: A, aminopeptidase; C, carboxypeptidase; D, dipeptidase; P, proteinase. Class indicates the class of the protease: A, asparatyl protease; M, metalloprotease; S, Serine protease; T, thiol protease. ^2^ Abundancies represent the sum of both the proenzyme and active enzymes where relevant. ^3^ Cathepsin B can exhibit both endoproteinase and carboxy-dipeptidase activities.

**Table 3:** List of 26 protease inhibitors identified in the proteome of at least 2/3 of the HM samples. Average ± St.dev. indicate average relative abundance +/− standard deviation in% of total protein. The type of inhibitor indicates the typical type of protease that is inhibited by the inhibitor (serine protease inhibitor, thiol = thiol protease inhibitor, metallo = metalloenzyme inhibitor). The order of the inhibitors is sorted from most to least abundant, based on the average of the relative abundance.

Protein ID ^1^	Gene	Inhibitor Name	Average ± St.dev. (in%)	Class of Protease Inhibitor
P29622	SERPINA4	Kallistatin	1.39 ± 2.23	S
P01011	SERPINA3	α-1-antichymotrypsin	0.54 ± 0.47	S
P01009	SERPINA1	α-1-antitrypsin	0.40 ± 0,12	S
P01034	CST3	Cystatin-C	0.19 ± 0.07	T
P01033	TIMP1	Metalloproteinase inhibitor 1	0.11 ± 0.05	M
P01042	KNG1	Kininogen-1	0.10 ± 0.03	T
P01008	SERPINC1	Antithrombin-III	0.06 ± 0.04	S
P05155	SERPING1	Plasma protease C1 inhibitor	0.05 ± 0.02	S
P01023	A2M	α-2-macroglobulin	0.04 ± 0.02	S, T, M ^2^
P08697	SERPINF2	α-2-antiplasmin	0.04 ± 0.01	S
P05067	APP	Amyloid-beta precursor protein	0.02 ± 0.02	S
P30086	PEBP1	Phosphatidylethanolamine-binding protein 1	0.02 ± 0.02	S
P02760	AMBP	Protein AMBP	0.02 ± 0.01	S
P04080	CSTB	Cystatin-B	0.02 ± 0.01	T
P19827	ITIH1	Inter- α -trypsin inhibitor heavy chain H1	0.01 ± 0.02	S
P35237	SERPINB6	Serpin B6	0.01 ± 0.01	S
P19823	ITIH2	Inter- α -trypsin inhibitor heavy chain H2	0.01 ± 0.01	S
Q99727	TIMP4	Metalloproteinase inhibitor 4	0.01 ± 7.89 × 10^−3^	M
P05546	SERPIND1	Heparin cofactor 2	0.01 ± 6.68 × 10^−3^	S
Q14508	WFDC2	WAP four-disulfide core domain protein 2	0.01 ± 8.06 × 10^−3^	S, T
P30740	SERPINB1	Leukocyte elastase inhibitor	<0.01	S
Q99574	SERPINI1	Neuroserpin	<0.01	S
Q06481	APLP2	Amyloid-beta precursor-like protein 2	<0.01	S
P20810	CAST	Calpastatin	<0.01	T
P05154	SERPINA5	Plasma serine protease inhibitor	<0.01	S
O95980	RECK	Reversion-inducing cysteine-rich protein with Kazal motifs	<0.01	S

^1^ According to uniprot.org. Class of protease inhibitor indicates the protease class inhibited by the inhibitor: A, asparatyl protease; M, metalloprotease; S, serine protease; T, thiol protease. ^2^ The A2M proteases can inhibit all four classes of proteinases via its unique trapping mechanism.

## Data Availability

The data presented in this study are available on request from the corresponding author. The data are not publicly available as the sample collection is still on-going and are thus not finally analyzed at this point in time.

## Supplementary Materials

The following supporting information can be downloaded at: https://www.mdpi.com/article/10.3390/nu15194169/s1, Table S1: Provides additional data about mothers 1 to 6 in study.
