# Supplementary material for: Fingerprinting of Proteases, Protease Inhibitors and Indigenous Peptides in Human Milk
Source: Nutrients. 2023 Sep 27;15(19):4169. doi: 10.3390/nu15194169 (PMC10574734; doi:10.3390/nu15194169)
Supplement: Supplementary file 1 [file nutrients-15-04169-s001.zip › nutrients-2597698-supplementary.pdf]

Supplementary table 1. : Provides additional data about mothers 1 to 6 in study.

ID	Age	Parity	Birth	Gestational age (w+d)
1	35	1	Vaginal	41+1
2	32	2	Vaginal	40+0
3	31	1	Vaginal	41+3
4	32	1	Vaginal	38+6
5	31	0	Vaginal	40+2
6	35	2	Vaginal	39+5
